# Macrolide-resistant *Mycoplasma pneumoniae* in adolescents with community-acquired pneumonia

**DOI:** 10.1186/1471-2334-12-126

**Published:** 2012-05-31

**Authors:** Naoyuki Miyashita, Yasuhiro Kawai, Hiroto Akaike, Kazunobu Ouchi, Toshikiyo Hayashi, Takeyuki Kurihara, Niro Okimoto

**Affiliations:** 1 Department of Internal Medicine I, Kawasaki Medical School, 2-1-80 Nakasange, Kita-ku, Okayama, 700-8505, Japan; 2 Department of Pediatrics, Kawasaki Medical School, 577 Matsushima, Kurashiki, 701-0192, Japan

**Keywords:** *Mycoplasma pneumoniae*, Macrolide-resistant, Community-acquired pneumonia, 23S rRNA

## Abstract

**Background:**

Although the prevalence of macrolide-resistant *Mycoplasma pneumoniae* isolates in Japanese pediatric patients has increased rapidly, there have been no reports concerning macrolide-resistant *M. pneumoniae* infection in adolescents aged 16 to 19 years old. The purpose of this study was to clarify the prevalence and clinical characteristics of macrolide-resistant *M. pneumoniae* in adolescent patients with community-acquired pneumonia.

**Methods:**

A total of 99 cases with *M. pneumoniae* pneumonia confirmed by polymerase chain reaction (PCR) and culture were analyzed. Forty-five cases were pediatric patients less than 16 years old, 26 cases were 16 to 19-year-old adolescent patients and 28 cases were adult patients. Primers for domain V of 23S rRNA were used and DNA sequences of the PCR products were compared with the sequence of an *M. pneumoniae* reference strain.

**Results:**

Thirty of 45 pediatric patients (66%), 12 of 26 adolescent patients (46%) and seven of 28 adult patients (25%) with *M. pneumoniae* pneumonia were found to be infected with macrolide-resistant *M. pneumoniae* (MR patients). Although the prevalence of resistant strains was similar in pediatric patients between 2008 and 2011, an increase in the prevalence of resistant strains was observed in adolescent patients. Among 30 pediatric MR patients, 26 had an A-to-G transition at position 2063 (A2063G) and four had an A-to-G transition at position 2064 (A2064G). In 12 adolescent MR patients, 10 showed an A2063G transition and two showed an A2064G transition, and in seven adult MR patients, six showed an A2063G transition and one showed an A2064G transition.

**Conclusions:**

The prevalence of macrolide-resistant *M. pneumoniae* is high among adolescent patients as well as pediatric patients less than 16-years old. To prevent outbreaks of *M. pneumoniae* infection, especially macrolide-resistant *M. pneumoniae*, in closed populations including among families, in schools and in university students, physicians should pay close attention to macrolide-resistant *M. pneumoniae*.

## Background

*Mycoplasma pneumoniae* is a common causative pathogen of respiratory infections in children and adults, accounting for as many as 10-30% of all cases of community-acquired pneumonia (CAP) [1]. *M. pneumoniae* pneumonia is specified for weekly reporting by specially designated sentinel clinics in accordance with the Japanese Infectious Diseases Control Law. Between 2001 and 2010, the average number of cases of *M. pneumoniae* pneumonia per sentinel clinic per year was 16.2 [2]. In 2011, especially the second half of the year, an epidemic of *M. pneumoniae* infection occurred throughout Japan and the incidence was the highest observed during the past decade, with the number of cases per sentinel clinic reaching 36.3 per year [2]. Many outbreaks of *M. pneumoniae* have occurred in closed populations including among families, in high schools, and in university students. During 2010 and 2011, a similar surge in *M. pneumoniae* infections was described in other countries especially in northern Europe [3-11].

Although *M. pneumoniae* pneumonia is usually mild in severity, macrolide-resistant *M. pneumoniae* with mutations in the 23S rRNA gene has emerged in pediatrics patients with CAP [12,13]. Recently, the prevalence of macrolide-resistant *M. pneumoniae* isolates in Japanese pediatric patients has increased sharply [14]. More than 60% of *M. pneumoniae* strains in pediatric patients showed high resistance to 14- and 15-membered ring macrolides with MICs greater than or equal to 32 mg/L [14]. In contrast to pediatric patients, the prevalence of macrolide-resistant *M. pneumoniae* infection in adult patients is low [15]. Previous reports were limited to pediatric patients less than 16 years old or adults (≥20 years old), and there are no reports investigating high school-aged patients (16 to 18-years old) [12,13,15-18]. Several studies to determine the influence of age in CAP patients indicated that *M. pneumoniae* pneumonia is significantly more common in younger patients and especially in the 10–20-year-old age group [1]. The purpose of this study was to clarify the prevalence and clinical characteristics of macrolide-resistant *M. pneumoniae* in adolescents, especially high school-aged patients, with CAP.

## Methods

### Patients

This study was conducted as a part of CAP studies that investigated the prevalence and clinical features of atypical pneumonia and evaluated the usefulness of diagnostic methods for the diagnosis of this condition. All patients with CAP who visited 12 institutions participating in the Atypical Pathogen Study Group from January 2008 to December 2011 were enrolled in this study. The diagnosis was based on clinical signs and symptoms of lower respiratory tract infection (cough, fever, productive sputum, dyspnea, chest pain, or abnormal breath sounds) and the presence of new infiltrates on chest radiographs that were at least segmental and were not caused by preexisting or other known causes. Informed consent was obtained from all patients; the study protocol was approved by the Ethics Committee at Kawasaki Medical School.

### Microbiological laboratory tests

Microbiological tests, such as Gram stain, cultures, real-time polymerase chain reaction (PCR), urinary antigen tests and serological tests, were performed as described previously [19]. Nasopharyngeal swab specimens were obtained from all patients and, if pleural fluid and sputum were available, a Gram stain test and a quantitative culture were obtained. Blood cultures were obtained from all adolescent and adult patients. Sputum data were only evaluated when the Gram stain test revealed numerous leukocytes (>25 in a × 100 microscopic field) and few squamous epithelial cells (<10 in a × 100 microscopic field). Invasive methods, such as bronchoscopic examination, were employed to obtain specimens in some patients after full explanation of the procedures. A bronchoscopic examination was undertaken for clinical indications. These specimens were also used for culturing and PCR. Cultures for *M. pneumoniae* and *Legionella* species were performed on pleuropneumonia-like organism broth (Difco, Detroit, MI, USA) and buffered charcoal-yeast extract alpha agar, respectively. Cultures for *Chlamydophila pneumoniae* and *C. psittaci* were performed using cycloheximide-treated HEp-2 cells grown in a 24-well cell culture plate. All specimens were examined twice. Culture confirmation was done by fluorescent-antibody staining with *C. pneumoniae* and *C. psittaci* species-specific and genus-specific monoclonal antibodies. The target DNA sequences for PCR were a region of the 53-kDa gene for *C. pneumoniae*, the major outer membrane gene for *Chlamydia*, the P1 cytadhesin gene for *M. pneumoniae*, and the nucleotide sequence of the 5S-ribosomal DNA for *Legionella*. DNA was extracted from respiratory samples using a QIAamp DNA Mini Kit (QIAGEN K. K., Tokyo, Japan) in accordance with the manufacturer’s instructions. The assays were performed as described previously [19]. Nasopharyngeal swab specimens were also tested for influenza A and B viruses by a direct enzyme immunoassay.

Paired serum samples were collected at intervals of at least 4 weeks after onset. Complement fixation (CF) tests were done in all patients for antibodies to influenza A and B viruses, adenovirus, respiratory syncytial virus, cytomegalovirus, and parainfluenza virus types 1, 2, and 3. Antibodies against *M. pneumoniae* were measured with the use of a particle agglutination (PA) test (Serodia-Myco II kit, Fujirebio, Tokyo, Japan), *Legionella* species by a microagglutination test (detection of *L. pneumophila* serogroups 1 ~ 6, *L. bozemanii*, *L. dumoffii*, *L. gormanii*, and *L. micdadei*), and *Coxiella burnetii* by an indirect immunofluorescence test. A microimmunofluorescence test was used for the titration of IgG and IgM antibodies against chlamydial species using formalinized elementary bodies of *C. pneumoniae* KKpn-15, *C. trachomatis* L2/434/Bu, and *C. psittaci* Budgerigar-1 strains as antigens. Rheumatoid factors were absorbed with GullSORB (Meridian Bioscience Inc., OH, USA) before IgM titration. In addition to serology, culturing, and/or PCR, urinary antigen tests (Binax NOW, Binax Inc. Portland, ME, USA) for *S. pneumoniae* and *L. pneumophila* were performed in adolescent and adult patients.

### Criteria for the determination of microbial etiology

The microbial etiology was classified as "definitive", "presumptive", or "unknown" as reported previously [19]. A definitive etiology was defined if one of the following conditions was present: (1) blood or pleural fluid cultures yielding the presence of bacterial or fungal pathogen; (2) urinary antigen test results positive for *L. pneumophila* or *S. pneumoniae*; (3) respiratory specimen culture or PCR results positive for *M. pneumoniae, C. psittaci* or *Legionella* species; (4) nasopharyngeal antigen test results positive for influenza A and B viruses; (5) a fourfold increase in the antibody titer for viruses, *M. pneumoniae* (to ≥1:160), *Legionella* species (to ≥1:128), *C. burnetii*, or *Chlamydia* species (IgM or IgG); or (6) a single increase in IgM titer for *Chlamydia* species ≥1:32. A presumptive etiology was considered if any of the following conditions were present: (1) an organism showing heavy (≥10^7^ cfu/mL) or moderate (10^6^ cfu/mL) growth of a predominant bacterium on a sputum culture in combination with Gram stain findings; (2) any microorganism isolated from bronchoscopic specimens when its concentration reached ≥10^5^ cfu/mL in quantitative culture; (3) an antibody titer of ≥1:320 for *M. pneumoniae* in either an acute-phase or convalescent-phase serum sample; (4) an antibody titer of ≥1:256 for *Legionella* species in either an acute-phase or convalescent-phase serum sample; or (5) respiratory specimens culture of PCR results positive for *C. pneumoniae*. An unknown etiology was considered if any of the following conditions were present: (1) respiratory specimens culture results were “normal flora”; (2) an organism showing light growth on a sputum culture; or (3) cases not fulfilling any of the above conditions.

### Detection of point mutations associated with resistance in domain V of 23S rRNA

A search for mutations at sites 2063, 2064, and 2617 in the *M. pneumoniae* 23S rRNA domain V gene region was performed using a direct sequencing method in samples with a positive PCR result, as reported previously [12,15,18]. Specifically, nested PCR was performed using a thermal cycler (PCR Thermal Cycler Dice Gradient, Takara Bio, Inc., Shiga, Japan) with primers (Sigma-Aldrich, Japan), Taq polymerase (Takara Ex *Taq* Version; Takara Bio, Inc.), and extracted DNA. The PCR products were purified using a QIAquick PCR Purification Kit (QIAGEN). The purified products were electrophoresed in a 3% Nusieve 3:1 agarose gel (Lonza) and, after the single band was confirmed, labeled using a BigDye Terminator V3.1 cycle sequencing kit (Applied Biosystems) and applied to an ABI Prism 3130x1 Genetic Analyzer (Applied Biosystems) in accordance with the manufacturer’s instructions. The presence or absence of gene mutations at each site was determined by reading using a sequence scanner (Applied Biosystems).

### Minimum inhibitory concentrations

The minimum inhibitory concentrations (MICs) of three agents for *M. pneumoniae* isolates were determined using microdilution methods with PPLO broth, as reported previously [12,15]. These agents were erythromycin, minocycline, and levofloxacin. *M. pneumoniae* strain M129 was used as a control. Serial twofold dilutions of antibiotics prepared in PPLO broth containing 10^4^ to 10^5^ CFU/mL of *M. pneumoniae* were placed in 96-well microplates [12,15]. The microplates were sealed with adhesive sheets and incubated at 37°C. The MIC was determined as the lowest concentration of antimicrobial agent at which the color of the control medium changed.

### Statistical analysis

Statistical analysis was performed using Stat View version 5.0. (SAS Institute Inc, Cary, NC, USA). The incidence of clinical findings was analyzed using Fisher's Exact test, and laboratory data were compared using Student's *t* test.

## Results

### Prevalence of macrolide-resistant M. pneumoniae in different age groups

During the study period, 1060 CAP cases were enrolled in this study. A total of 423 cases were pediatric patients less than 16 years old, 124 cases were 16 to 19-year-old adolescent patients, and 513 cases were adult patients. A microbiological diagnosis was established in 56% of pediatric patients, 59% of adolescent patients, and 54% of adult patients. The most common pathogens were *M. pneumoniae* (23%) followed by *Haemophilus influenzae* (15%) and *S. pneumoniae* (8%) in pediatric patients, *M. pneumoniae* (29%) followed by *S. pneumoniae* (14%) and *H. influenzae* (10%) in adolescent patients, and *S. pneumoniae* (26%) followed by *M. pneumoniae* (10%) and *H. influenzae* (7%) in adult patients.

Of all CAP cases, 99 cases were positive for *M. pneumoniae* in culture and/or by the PCR method. Of these, all cases were PCR positive, 40 cases were culture positive and 85 cases demonstrated positive serological results. No other microorganisms were detected in these cases. Forty-five cases were pediatric patients, 26 cases were adolescent patients and 28 cases were adult patients.

**Figure 1:**
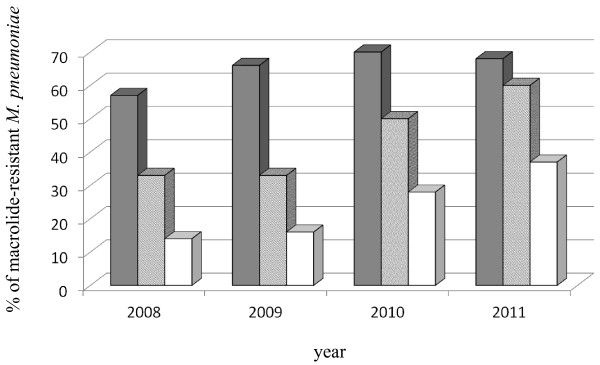
**The prevalence of macrolide-resistant *M. pneumoniae *in different age groups between 2008 and 2011**. Although the prevalence of resistant strains was similar in pediatric patients between 2008 and 2011, an increase in the prevalence of resistant strains was observed in adolescent patients. Shaded bar: pediatric patients less than 16 years old, hatched bar: 16 to 19-year-old adolescent patients, open bar: adult patients.

Thirty of 45 pediatric patients (66%), 12 of 26 adolescent patients (46%), and seven of 28 adult patients (25%) with *M. pneumoniae* pneumonia were found to be infected with macrolide-resistant *M. pneumoniae* (MR) patients. The prevalence of macrolide-resistant *M. pneumoniae* in different age groups from 2008 to 2011 is presented in Figure 1. Among adolescent patients, macrolide-resistant *M. pneumoniae* was identified in two of six patients in 2008, two of six patients in 2009, two of four patients in 2010 and six of 10 patients in 2011.

### Patient characteristics

Table 1 shows the characteristics of MR and macrolide-sensitive *M. pneumoniae* (MS) patients in different age groups. As reported by Lucier et al. [20], Okazaki et al. [13], and Morozumi et al. [14], an A-to-G transition or A-to-C transversion at positions 2063 or 2064 in domain V on the 23 S rRNA gene results in resistance to macrolide antibiotics. Among 30 pediatric MR patients, 26 had an A-to-G transition at position 2063 (A2063G) and four had an A-to-G transition at position 2064 (A2064G). In 12 adolescent MR patients, 10 showed an A2063G transition and two showed an A2064G transition, and in seven adult MR patients, six showed an A2063G transition and one showed an A2064G transition. No mutations at site 2617 in domain V of the 23 S rRNA gene were observed. In terms of clinical symptoms and laboratory findings, no significant differences were observed between MR patients and MS patients in different age groups.

**Table 1:** **Characteristics of patients with macrolide-resistant (MR) and macrolide-sensitive (MS) *Mycoplasma pneumoniae *pneumonia in different age groups**

**Characteristic**	**Pediatric patients**		**Adolescent patients**		**Adult patients**	
	**MR**	**MS**	**MR**	**MS**	**MR**	**MS**
Number	30	15	12	14	7	21
Age range, years	1–15	1–15	16–19	16–19	20–42	28–45
Male:female	16:14	7:8	6:6	8:6	4:3	11:10
Mutation site in 23S rRNA ^a^						
A2063G	26		10		6	
A2064G	4		2		1	
No. of patients prescribed macrolides (%)	23 (76)	15 (100)	9 (75)	9 (64)	5 (71)	6 (28)
Clarithromycin	12	10	5	4	2	2
Azithromycin	11	5	4	5	3	4
No. of patients with a change of prescription after macrolide administration (%)	20 (66)	0	7 (58)	3 (21)	3 (42)	4 (19)
Minocycline	16		5	3	1	1
Quinolones	4 ^b^		2	0	2	3
Clinical symptoms and laboratory findings at the first examination						
Cough	30	15	12	14	7	21
Sputum	23	11	7	8	4	12
Fever, temperature ≥ 38°C	26	14	11	13	7	21
Respiratory rate, > 30/min	2	1	0	0	0	0
White Blood Cell, mean (/uL)	6,410	6,520	6,810	7,010	6,620	6,710
C-reactive protein, mean (mg/dL)	2.1	1.9	3.1	3.3	3.7	3.5

^a^ A2063G: A-to-G transition at position 2063 in domain V on the 23S rRNA gene.

A2064G: A-to-G transition at position 2064.

^b^ Tosufloxacin used.

### Minimum inhibitory concentrations

Twenty-two isolates of *M. pneumoniae* with a mutation of the 23 S rRNA gene showed resistance to erythromycin with a MIC of 128 to >128 mg/L (MIC_90_ of >128 mg/L), whereas the other 18 isolates without a mutation of the 23 S rRNA gene showed susceptibility to erythromycin with a MIC of 0.00195 to 0.0078 mg/L (MIC_90_ of 0.0078 mg/L). The MICs for minocycline and levofloxacin for macrolide-resistant isolates were equal to the MIC for susceptible isolates at 0.25 to 2 mg/L (MIC_90_ of 1 mg/L) and 0.25 to 1 mg/L (MIC_90_ of 0.5 mg/L), respectively.

## Discussion

Macrolides are generally considered to be the first-choice agents for the treatment of *M. pneumoniae* infection. Tetracyclines and fluoroquinolones are effective in the treatment of *M. pneumoniae* infection, but the administration of these agents to children is not recommended because of their toxicity. Tetracyclines have the potential to repress bone growth, cause permanent gray-brown discoloration of the teeth, and enamel hypoplasia when given during tooth development. The clinical importance of fluoroquinolones has not been demonstrated because they have been known to cause cartilage erosion in young animals. Thus, these agents should be avoided when alternatives can be used. In contrast to pediatric patients, fluoroquinolones and tetracyclines are usually administered to adult patients with respiratory tract infections. These agents demonstrate good clinical efficacy against macrolide-resistant *M. pneumoniae* infection. Adolescent patients usually visit a general physician not a pediatrician and fluoroquinolones and tetracyclines may often be administered against respiratory tract infections. General physicians may readily treat respiratory infections using these agents in adult and adolescent patients. Thus, the investigation of macrolide-resistant *M. pneumoniae* infections has been carried out only among the pediatricians in Japan.

In a previous study, we first reported macrolide-resistant *M. pneumoniae* infections in adults (≥20 years old) in Japan [15]. In the present study, the prevalence of macrolide-resistant *M. pneumoniae* was similar between pediatric and adolescent patients especially in 2011. Point mutations in domain V of 23 S rRNA were also identical between these two groups. Physicians should pay attention to macrolide-resistant *M. pneumoniae* not only in children but also in adolescent patients, especially those of high-school age.

In 2009, the first adult case of CAP caused by macrolide-resistant *M. pneumoniae* was described in Japan [21]. We have been investigating the prevalence of macrolide-resistant *M. pneumoniae* in both children and adults since 2005, and we identified among adult patients, one of seven patients in 2008, one of six patients in 2009, two of seven patients in 2010, and three of eight patients in 2011 were infected with macrolide-resistant *M. pneumoniae*[15]. Thus, the prevalence of macrolide-resistant *M. pneumoniae* may be increasing in adult patients.

Macrolide-resistant *M. pneumoniae* is also emerging in other several countries especially in children [5,22-26]. A recent study in China has shown macrolide resistance in 69% of *M. pneumoniae* isolates from adolescent and adult patients [26]. Thus, monitoring of *M. pneumoniae* strains seems to be necessary in order to recognize early changes in the antibiotic resistance pattern of this important agent of human respiratory tract infections.

Our study had limitations; we evaluated PCR-positive patients (of these, 40 cases were culture positive). As reported by several researchers, an A2063G transition and an A2064G transition in domain V of the 23 S rRNA gene resulted in resistance to macrolide antibiotics [13]‐[15,20]. Among the 49 MR patients studied, 32 had A2063G and seven had A2064G. Most of these isolates were highly resistant to 14- and 15-membered ring macrolides with MICs greater than or equal to 32 μg/mL [13-15,20]. Thus, these data indicate that our PCR-positive patients with point mutations at positions 2063 or 2064 in domain V on the 23 S rRNA gene were resistant to macrolides. Furthermore, the number of patients characterized as adolescents was very low to clarify the prevalence of resistance in this age group. A large-scale surveillance study to investigate the frequency of macrolide-resistant *M. pneumoniae* cases is needed.

## Conclusions

The prevalence of macrolide-resistant *M. pneumoniae* is high among adolescent patients as well as pediatric patients less than 16-years old. To prevent outbreaks of *M. pneumoniae* infection, especially macrolide-resistant *M. pneumoniae*, in closed populations including among families, in schools, and in university students, physicians should pay careful attention to the potential occurrence of infections involving macrolide-resistant *M. pneumoniae*.

## Competing interests

The authors declare that they have no competing interests.

## Authors’ contributions

NM, KO and NO conceived the study and participated in its design and coordination. NM, YK, HA, TH, TK and the Atypical Pathogen Study Group members collected and managed the data, including quality control, and carried out the microbiological laboratory tests. NM, KO and NO drafted the manuscript, and all authors contributed substantially to its revision. All the authors read and approved the final manuscript.

## Pre-publication history

The pre-publication history for this paper can be accessed here:

http://www.biomedcentral.com/1471-2334/12/126/prepub
